# Wilkie’s Weight Loss Wonder: A Case Series

**DOI:** 10.51894/001c.13485

**Published:** 2020-10-30

**Authors:** Akhlema Haidar, Alexandra Davies, Afzal Hussain, Samuel Gregerson, Dheeraj Thammineni, Johnathon Markus

**Affiliations:** 1 Internal Medicine McLaren Macomb; 2 General Surgery McLaren Macomb; 3 Michigan State University; 4 Gastroenterology McLaren Macomb

**Keywords:** bowel obstruction, weight loss, wilkie’s syndrome, superior mesenteric artery syndrome

## Abstract

**INTRODUCTION:**

Wilkie’s Syndrome, also known as Superior Mesenteric Artery Syndrome (SMAS), is a rare cause of bowel obstruction that can contribute to vague abdominal symptoms on clinical presentation. This syndrome occurs when the aortomesenteric angle decreases, compressing the third portion of the duodenum between the aorta and the superior mesenteric artery. An acute decrease in the mesenteric fat pad cushion between these two blood vessels is the primary etiology, although other causes (e.g., anatomical, postoperative, functional, and pubescent etiologies) have also been described.

**CASE PRESENTATION:**

In the present cases, 2 females with a common history of recent weight loss presented to our institution with similar symptoms of abdominal pain, nausea and vomiting. Each patient was subsequently diagnosed with SMAS following imaging studies. Both patients experienced successful resolution of symptoms with conservative nutritional management.

**DISCUSSION:**

Common presenting complaints of SMAS include nausea, vomiting, early satiety and postprandial pain. These symptoms overlap with other gastrointestinal disorders (i.e., mesenteric ischemia, intestinal volvulus, peptic ulcer disease) making diagnosis difficult. SMAS can be identified through imaging modalities including barium studies and computer tomography. First line therapies typically include conservative nutritional support and promotion of weight gain. If conservative therapies fail, various surgical procedures can be pursued. Delayed diagnosis can lead to further pathological sequelae, including duodenal compromise, ischemia and necrosis. As the syndrome progresses, success of conservative nutritional support is less likely, and surgical correction becomes increasingly necessary.

**CONCLUSION:**

Therefore, a clinical goal for SMAS should include as swift a recognition and diagnosis as possible.

## INTODUCTION

Wilkie’s Syndrome, also known as Superior Mesenteric Artery Syndrome (SMAS), is a rare cause of bowel obstruction that can lead to vague abdominal symptoms on clinical presentation. SMAS occurs when the aortomesenteric angle (i.e., normally 38-65 degrees) decreases, compressing the third portion of the duodenum between the aorta and the superior mesenteric artery.^1^ Most commonly, an acute decrease in the mesenteric fat pad cushion between these two blood vessels is the primary etiology.^1^ Abnormal gastrointestinal anatomy, corrective spinal surgery, and pediatric growth spurts have all been described causing SMAS as well.^2–6^

Conditions causing rapid weight loss (e.g., anorexia nervosa, malabsorption, and cancer) can each decrease the fat pad cushion, leading to SMAS.^2^ Common presenting complaints include less specific gastrointestinal obstruction symptoms, such as nausea, vomiting, early satiety (i.e., “feeling full”) and postprandial (i.e., after meal) pain.^1^ These initial complaints can lead to further cyclical food aversion, poor fluid and food intake, and weight loss that further worsen symptoms.^1^

Once recognized, first line treatment strategies include conservative nutritional support.^3^ Conservative nutritional support consists of intravenous fluids, nasogastric tube placement for decompression, total parenteral nutrition and anti-emetic medications.^1^ If conservative measures fail (i.e., symptoms remain severe) or the duodenum is still compromised, surgical procedures (e.g., laparoscopic duodenojejunostomy, gastrojejunostomy, Strong’s procedure) are routinely considered.^1^

SMAS is rare, with a reported prevalence of less than 0.3% among adults.^4^ Furthermore, the varying etiology and overlapping symptomatology with other gastrointestinal pathologies (i.e., gastroesophageal reflux disease, mesenteric ischemia, biliary colic, peptic ulcer disease, intestinal volvulus) can make diagnosing SMAS quite difficult.^4^ Maintaining a high clinical index of suspicion can lead to earlier diagnosis, allowing for conservative treatments to be more effective. The following outlines two recent cases of SMAS diagnosed at the authors’ institution that were successfully treated with conservative management.

### Case Reports:

A female in her early to mid-20’s presented to our McLaren Macomb hospital’s emergency department with a chief complaint of intractable abdominal pain. She had woken up in the middle of the night two nights earlier with stomach pain and claimed she could feel “fluid moving around her stomach” when changing positions. She reported four-to-five episodes of projectile vomiting, which provided temporary relief, as did sitting upright. Eating and drinking reportedly precipitated her abdominal pain and vomiting.

This patient denied radiation of the pain elsewhere in the body, but described the stomach pain as a constant, generalized “fullness” She reported a significant weight loss of 15-to-20 pounds during the preceding three weeks. The patient denied fevers, diarrhea, recent sick contacts or travel.

Her past medical history was significant for anxiety and intravenous drug use. The patient had undergone multiple drug rehabilitation periods and admitted to last using intravenous drugs during the past weekend. She denied alcohol use but has smoked one pack of cigarettes/day since she was 17 years old. When asked about her diet, the patient said, “I know I should be eating better and more often.” In this case, the patient’s weight loss was believed to be multifactorial in origin (i.e. poor nutrition with recent illicit drug and tobacco use).

On physical exam, the patient was anxious and restless, answering questions while pacing throughout the room. Vitals remained within normal limits during hospital stay, with a relevant unhealthy body mass index (BMI) of 17.2. Other pertinent findings included abdominal distension, diffuse abdominal tenderness to palpation and a positive succussion splash (i.e., sloshing sound heard through stethoscope during abdominal auscultation).

**Image 1:**
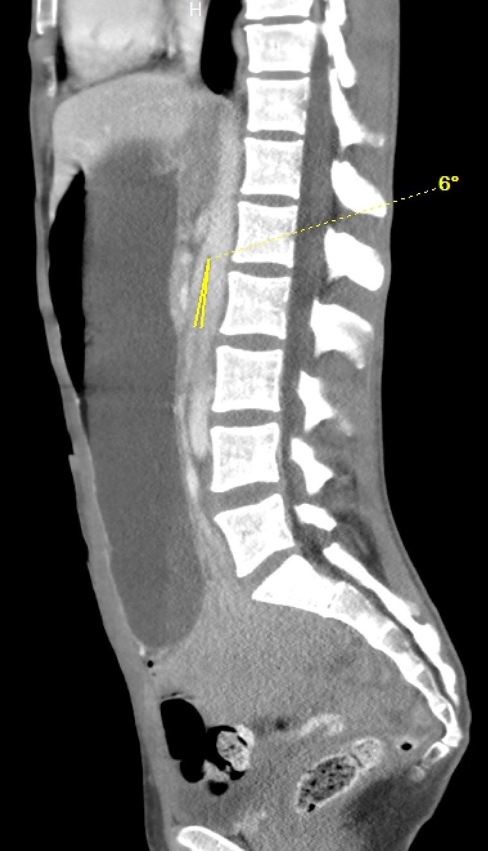
A computer tomography image of the abdomen and pelvis with intravenous contrast showed a significantly fluid distended stomach and SMA angle of 6 degrees (yellow arrow).

**Image 2:**
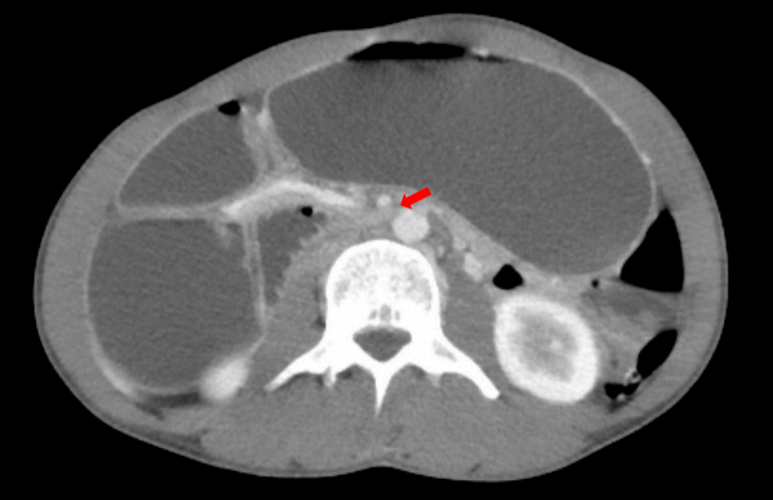
A computer tomography image of the abdomen and pelvis with intravenous contrast showed the duodenum as it passes between the SMA and aorta which demonstrated significant narrowing (red arrow), consistent with SMAS.

On hospital Day 1, an esophagogastroduodenoscopy (EGD) with nasogastric tube placement was completed with removal of approximately two liters of chyme (i.e., acidic gastric fluid). Initial computer tomography was reviewed by Radiology, and SMAS was diagnosed (Images 1 and 2). After a multidisciplinary review consisting of the case’s internal medicine, gastroenterology and general surgery teams, the patient was transferred to a tertiary care center where a gastrostomy-jejunostomy tube was placed. She was started on tube feedings and reported improvement of symptoms at follow-up one month later.

In the second case, a female in her early forties with no medical history presented with acute onset of diffuse abdominal pain, vomiting, and diarrhea over the previous three days. The patient endorsed multiple years of intermittent symptoms of nausea and abdominal pain alleviated by emesis (vomiting), but never to this severity. She reported a weight loss of approximately 15 pounds during the last two weeks.

**Image 3:**
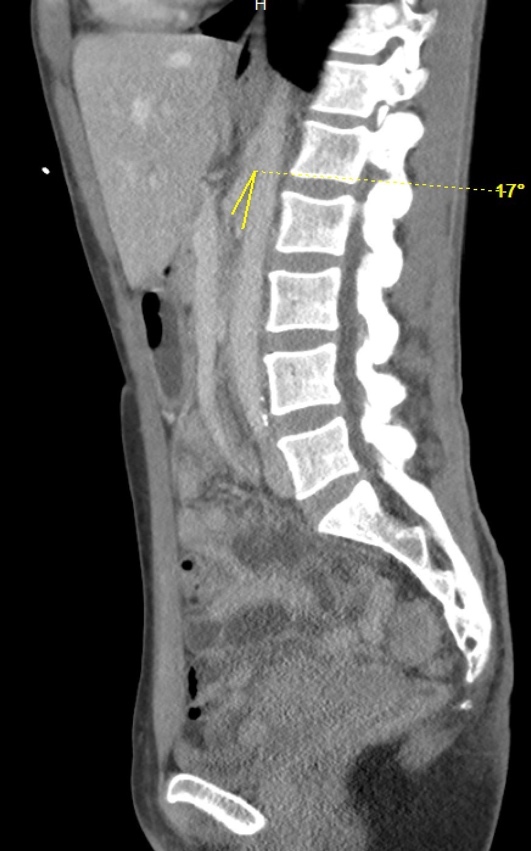
A computer tomography image of the abdomen and pelvis with intravenous contrast showed a SMA angle of 17 degrees (yellow arrow).

**Image 4:**
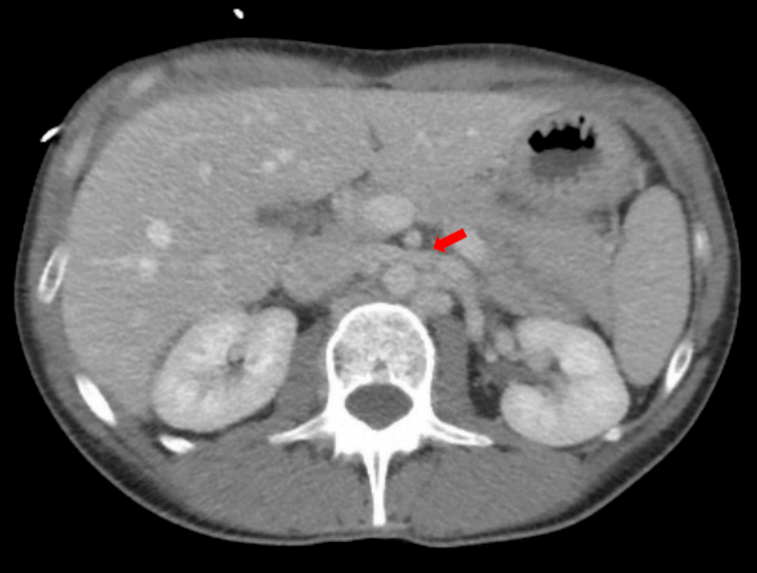
A computer tomography image of the abdomen and pelvis with intravenous contrast showed the duodenum as it passes between the SMA and aorta which demonstrated significant narrowing (red arrow), consistent with SMAS.

Pertinent findings on physical exam included mid-epigastric abdominal pain with an underweight BMI of 18.6. Computer tomography of the abdomen and pelvis with intravenous contrast provided a radiological diagnosis of SMAS (Images 3 and 4).

On hospital Day 4, the patient had a peripherally inserted central catheter (PICC) line placed and was started on total parenteral nutrition (TPN) due to her inability to tolerate oral feeds. Throughout her two-week hospital course, the patient’s symptoms improved, and she was later able to supplement her nutrition with oral feeds and mirtazapine to decrease her nausea and vomiting.

## DISCUSSION

SMAS is a rare cause of partial or complete duodenal obstruction that can manifest with nonspecific abdominal symptoms. Rapid weight loss leading to a reduction in the mesenteric fat pad is a common cause of the syndrome. However, other conditions (i.e., anatomical, surgical, pubescent) may predispose aortomesenteric angle decreases as well.^2–6^

Congenital SMAS is very rare, with only a small number of patients being diagnosed in the medical literature. Congenital SMAS occurs due to an abnormal anatomical placement of the ligament of Treitz.^5^ The syndrome can also be a postoperative complication in corrective spinal surgery for scoliosis due to the acute vertical traction and stress placed on the spine, leading to abrupt aortomesenteric angle narrowing.^6^

Functional SMAS has been described, with one particular case caused by an inferior vena cava (IVC) filter that created a local inflammatory response, compressing the duodenum between the IVC and the superior mesenteric artery.^7^SMAS can be correlated with renal nutcracker syndrome, in which the left renal vein is obstructed by the same pathogenesis as SMAS.^8^ SMAS can even be seen in adolescents undergoing growth spurts, due to a significant increase in lean body mass, linear growth and loss of adipose tissues.^4^

SMAS can primarily be identified through imaging modalities such as barium studies and computer tomography. The first line therapy consists of conservative nutritional support and promotion of weight gain. This is achieved through a variety of methods, including intravenous fluids, nasogastric tube placement for decompression, total parenteral nutrition, and anti-emetic medications.^4^ If conservative therapy fails, various surgical procedures can be pursued.

Duodenojejunostomy is the surgical procedure of choice, with a success rate of 90%.^9^ Other procedures, such as a gastrojejunostomy and Strong’s procedure have also been used to treat SMAS.^9^ Since SMAS can also be caused by eating disorders (e.g., anorexia nervosa), some investigators have proposed that medical or surgical treatment be supplemented with psychological assessment to treat any underlying psychosocial conditions.^10^

## CONCLUSION

SMAS is a rare and challenging medical diagnosis due to its various etiologies and nonspecific symptoms that overlap with other bowel obstruction disorders. The combination of insidious onset and nonspecific symptoms can obscure and delay diagnosis. A delayed diagnosis can lead to further pathological sequelae, including duodenal compromise, ischemia and necrosis.

As the syndrome progresses, success of conservative nutritional support is less likely, and surgical correction becomes necessary. Therefore, a clinical goal for SMAS should include swift recognition and diagnosis. This case series highlights the importance of maintaining a high clinical suspicion of SMA Syndrome in a patient presenting with obstructive gastrointestinal symptoms combined with recent weight loss. A faster diagnosis and appropriate nutritional support may help patients avoid further disease complications and the need for surgical procedures.

The authors report no external funding source for this study.

The authors declare no conflict of interest.

Submitted for publication May 2020.

Accepted for publication June 2020.
